# Comprehensive Analysis of Storage Stability of Hong-Jam Under Various Conditions: Correlation Between Lipid Oxidation Factors and Alternative Quality Parameters

**DOI:** 10.3390/foods14091593

**Published:** 2025-04-30

**Authors:** Min-Seok Kim, Sang-Jin Ye, Ji-Hwan Roh, Hyun-Wook Choi, Moo-Yeol Baik

**Affiliations:** 1 Department of Food Science and Biotechnology, Institute of Life Science and Resources, Kyung-Hee University, Yong-in 17104, Republic of Korea; msyw617@khu.ac.kr (M.-S.K.); ysj7153@korea.kr (S.-J.Y.); kidroh@khu.ac.kr (J.-H.R.); 2 Department of Food and Nutrition, Jeonju University, Jeonju 55069, Republic of Korea

**Keywords:** Hong-Jam, storage stability, shelf life, lipid oxidation, correlation analysis

## Abstract

Hong-Jam is a functional food product made from freeze-dried silkworms on the seventh to eights day of the fifth Instar stage, which are steamed for 2 h prior to drying. This study evaluated the storage stability of Hong-Jam under varying temperatures, product forms, and packaging conditions. By using the correlation analysis, novel quality indices that may complement or replace traditional lipid-oxidation markers in edible-insect products were identified. Peroxide value (POV) and acid value (AV), which are factors of lipid oxidation, significantly increased under high-temperature conditions. Dependency of temperature (D_T_) analysis revealed that POV had a higher D_T_ than AV, and glass bottles exhibited higher D_T_ than polyethylene (PE) pouches. In the four-way analysis of variance (ANOVA) analysis, the Fisher’s F-statistic (F-value) for single effects of individual storage conditions was notably higher than the interaction effects. During storage, the moisture content increased and the pH decreased, while the color remained relatively stable. The results of the correlation analysis indicated that there was a significant correlation between moisture content and pH with lipid oxidation indices such as POV and AV. This suggests that these two factors could be used as complementary factors in quality assessments. In conclusion, this research provides foundational data that can be used to develop improved storage standards and quality assurance protocols for Hong-Jam and similar edible insect foods. These advancements will contribute to extending shelf life, enhancing consumer safety, and promoting the broader acceptance of edible insects in the food industry. Through a comprehensive understanding of the stability factors, this study supports the development of reliable quality management practices that align with the increasing consumer demand for sustainable and functional food options.

## 1. Introduction

Hong-Jam is a functional food that is made through the steam-drying of silkworms on the seventh to eighth day of the fifth Instar stage at 100 °C for 2 h, followed by freeze-drying. The silkworms used in the production of Hong-Jam are mature silkworms, which are silkworms that have fully grown and are just about to start spinning their cocoons, filled with silk glands rich in protein [1]. These mature silkworms are of nutritional value due to their high protein content, which includes essential amino acids, peptides, and bioactive compounds that contribute to their functionality as a nutrient-dense food source [2,3].

To maintain nutritional components and improve storage stability, silkworms are subjected to a drying process, which serves to extend their shelf life and preserve their bioactive properties [4,5]. Freeze-drying is used specifically for its ability to preserve the nutritional and structural integrity of the silkworm, minimizing the loss of heat-sensitive components such as vitamins, antioxidants, and functional peptides [3]. This method also preserves the light, crisp texture of the product, making it more palatable for consumers. However, hot-air drying poses significant problems due to the high temperatures, which denature and harden the silk proteins in the silkworm, making them difficult to consume [6]. The protein aggregation caused by hot air drying compromises the nutritional and sensory qualities of the final product, highlighting the need for alternative drying methods. To address these drawbacks, studies have evaluated the effects of different processing methods by classifying the silkworms as frozen, freeze-dried, and fresh prior to steaming and drying [7]. These investigations have shown that freeze-drying is effective in softening the silk proteins and maintaining the nutritional quality of the silkworms. In addition, different pre-treatments have been studied to understand the changes in the overall composition and functional properties of silkworms [8].

Although there is a growing global interest in edible insects as sustainable food sources due to their low environmental footprint, high protein efficiency, and potential to address food insecurity, recent research has primarily focused on their nutritional value and consumer acceptance [9,10,11]. However, there remains a critical gap in understanding how storage conditions and packaging affect the quality and stability of insect-based foods [12,13]. These factors are especially important for lipid- and protein-rich foods like Hong-Jam, where quality changes such as lipid oxidation, moisture uptake, and pH shifts can significantly impact shelf life and functionality [14,15,16].

In this study, we assessed the storage stability of Hong-Jam under various packaging materials and storage forms, primarily examining lipid oxidation through indicators such as peroxide value (POV) and acid value (AV). To further enhance the evaluation of Hong-Jam’s stability, we aimed to develop supplementary quality indicators alongside these traditional markers. By applying the Arrhenius equation, we analyzed the temperature dependence of lipid oxidation and subsequently derived the product’s shelf life, thereby providing a more comprehensive basis for assessing its overall storage stability.

Building on these findings, we investigated how multiple variables interact to influence Hong-Jam’s storage stability. Through correlation analyses and Principal component analysis (PCA), we clarified the relationships among these factors and quantified their respective impacts. Furthermore, we employed partial least squares regression (PLSR) and partial least squares discriminant analysis (PLS-DA) to develop and evaluate predictive models for POV and AV, thereby illustrating how specific packaging choices or product forms can directly affect Hong-Jam’s stability under different storage conditions. Through these integrated analytical approaches, this research ultimately seeks to establish more robust quality assurance protocols for Hong-Jam and similar edible insect foods, contributing to wider acceptance and commercialization of sustainable, functional food resources.

## 2. Materials and Methods

### 2.1. Materials

Hong-Jam, produced in June 2023, was provided by the National Institute of Agricultural Science. Chemicals were purchased from Samchun Chemicals Co. (Seoul, Republic of Korea).

### 2.2. Sample Preparation

To evaluate the stability of different product forms, Hong-Jam was used either as whole dried larva (raw) or as powder obtained by one-step fine grinding. The powder was produced with a stainless-steel blade grinder (model SFM-C353NK, Shinil Co., Suwon, Republic of Korea). It was not sieved or size-classified, because post-grinding fractionation would remove cuticular fragments and lipid-rich tissues, creating a composition no longer comparable with the intact larvae. Moreover, commercially marketed Hong-Jam powders are sold without mesh standardization. After preparation, each sample (30 g, w.b.) was packaged in either polyethylene pouches (150 mm × 40 mm × 220 mm; thickness, 0.10 mm; single-layer LLDPE) and glass bottles (250 mL; 70 mm × 143 mm) to assess packaging effects. All containers were wrapped in aluminum foil to block light and prevent photo-oxidation.

### 2.3. Storage Condition

The storage conditions for Hong-Jam were designed based on accelerated shelf life test [17,18]. Samples were stored at four different temperatures: 5, 25, 35, and 45 °C (±2 °C), representing cold, ambient, warm, and accelerated conditions, respectively. At the time of grinding and packaging, the relative humidity was 50–55% RH. After sealing, the packages were not reopened during the entire storage period, so the internal humidity was governed by the product’s initial moisture content rather than the external environment.

Quality factors of each sample, including moisture content, color stability, antioxidant capacity, microbial load, and physicochemical changes, were monitored at 4-week intervals over a 16-week period. These evaluations were designed to provide insights into degradation kinetics and identify critical factors affecting product stability.

### 2.4. Quality Factors

#### 2.4.1. Moisture Content

Moisture content was measured according to AOAC official methods [19]. Samples were weighed accurately before being dried in an oven at 105 °C until a constant weight was reached. The moisture content was calculated as the difference between the initial and final weights of the sample, expressed as a percentage of the initial weight.

#### 2.4.2. pH

The pH of the Hong-Jam samples was determined using a digital pH meter (FP20, Mettler Toledo, Switzerland). Before analysis, samples (1 g, d.b.) were ground to create a uniform mixture in 50 mL beaker with 20 mL distilled water. The homogenate was then filtered with a filter paper (Whatman filter paper Grade 2) to remove solid particles, ensuring accurate pH measurement of the liquid portion.

#### 2.4.3. Color

Color was measured using a colorimeter (CR-400, Minolta Co., Tokyo, Japan), which was calibrated with a standard white plate (L = 97.79, a = −0.38, b = 2.05) before each set of measurements.

#### 2.4.4. Peroxide Value (POV)

Samples (1 g, w.b.) were accurately weighed and dissolved in 25 mL of a mixture of acetic acid and chloroform (3:2, *v*/*v*), ensuring thorough mixing to achieve complete dissolution. Subsequently, 1 mL of a saturated potassium iodide solution was added, and the mixture was gently shaken and then left in the dark for 10 min to allow the reaction between iodine and peroxides. After the reaction period, 30 mL of distilled water was added to the mixture, which was then vigorously shaken to ensure proper mixing. The solution was titrated with 0.01 N sodium thiosulfate solution using 1 mL of freshly prepared starch solution as an indicator. The titration was continued until the blue color disappeared, indicating the endpoint. Blank tests were performed to calibrate the titration, accounting for any background peroxides or iodide present. The peroxide value was calculated using the following equation:

$$Peroxide\;value\;\left(\mathrm{meq}/\mathrm{kg}\right)=\frac{\left(a-b\right)\times f}{weight\;of\;sample\;\left(w.b.\right)}\times 10$$

*a*: consumption of 0.01 N sodium thiosulfate solution (mL)
*b*: blank
*f*: titer of 0.01 N sodium thiosulfate solution (= 1.0)

#### 2.4.5. Acid Value (AV)

Samples (5 g, w.b.) were accurately weighed and dissolved in 100 mL of a solvent mixture composed of ethanol and ether (1:2, *v*/*v*). The mixture was thoroughly mixed to ensure complete dissolution of the sample. The resulting solution was then titrated with a standardized 0.1 N KOH/ethanol aqueous solution using phenolphthalein as an indicator, which changes color at the endpoint, indicating neutralization of the free fatty acids. The acid value was calculated as the amount of KOH required to neutralize the free fatty acids in 1 g of the sample, using the following equation:

$$Acid\;value\;\left(KOH\;\mathrm{mg}/g\right)=\frac{5.611\times \left(a-b\right)\times f}{weight\;of\;sample\;\left(w.b.\right)}$$

*a*: consumption of 0.1 N ethanol aqueous potassium hydroxide solution (mL)
*b*: blank
*f*: titer of 0.1 N ethanol aqueous potassium hydroxide solution (= 1.0)

### 2.5. Determination of Dependency on Temperature (D_T_) and Shelf Life

This study investigated the temperature dependence of quality factors in Hong-Jam and analyzed the degradation reactions by dividing them into zero and first order. To analyze temperature effects on reaction rate constants, the Arrhenius equation was applied. The reaction rate constant (*k*) was determined through linear regression of experimental data, using the Arrhenius equation to relate rate constants to temperature conditions.

In order to predict the shelf life of Hong-Jam, experimental guidelines from the Korean Ministry of Food and Drug Safety were followed [20]. These guidelines were based on the use of POV threshold of less than 60 meq/kg and AV threshold of less than 5.0 mg/g, which were indicated as the acceptable limits for product quality and safety. The quality and safety limit period, or the time required for each quality factor to reach the specified threshold, was calculated under each storage condition. Furthermore, a safety factor of 0.7 was applied to the quality and safety limit period to account for potential variability during distribution and consumption, resulting in the final predicted shelf life. This adjustment ensures product safety even under slightly adverse conditions. The shelf life was determined using the following equation:

$$t_{n=0}=\frac{\left[A_{0}\right]-\left[A_{t}\right]}{k_{n=0}}$$

$$t_{n=1}=\frac{ln\left[A_{0}\right]-ln[A_{t}]\;}{k_{n=1}}$$

*t*: shelf life
*n*: reaction order
*A*_0_: value of quality factor at 0 week
*A*_t_: value of quality factor at t weeks
*k*: reaction rate constant

The activation energy ($E_{a}$) of the reactions was determined from the slope of the straight line in the Arrhenius plot, using the following Arrhenius equation:

$$k=Ae^{-\frac{E_{a}}{RT}}$$

$$E_{a}=\left|Slope\right|\times R$$

*k*: reaction rate constant
*A*: frequency factor
*E*_a_: activation energy (cal/mol)
*R*: gas constant (= 1.986 cal/mol K)
*T*: Absolute temperature (K)

The $Q_{10}$ value was determined using the following equation:

$$Ln\;Q_{10}=10\times E_{a}/R\times T(T+10)$$

*Q*_10_: sensitivity of responses to a 10 °C change in temperature
*E_a_*: activation engergy
*R*: gas constant

### 2.6. Statistical Analysis

All experiments were repeated at least three times and expressed as mean values with standard deviations. Multiple comparisons between means were performed using Tukey’s test at the 95% confidence level (*p* < 0.05). All statistical analyses were conducted using the SAS program (version 9.4; SAS Institute, Inc., Cary, NC, USA). Correlation analysis between two parameters was performed using Pearson’s test (SPSS Inc., Chicago, IL, USA).

Before analysis, all data were standardized to ensure comparability across variables. PCA was conducted using the *prcomp* function in R Studio (R version 4.4.1; R Foundation for Statistical Computing, Vienna, Austria) to identify key quality variables and explain variance among data points. Visualization of PCA results, including scree plots and biplots, was performed using the *factoextra* package to interpret the contributions of each variable to the principal components.

PLSR was applied to develop predictive models for lipid oxidation indicators, such as peroxide value (POV) and acid value (AV), based on supplementary parameters, including moisture content, pH, and color. This analysis was conducted using the *pls* package, with model performance evaluated through cross-validation. Metrics such as root mean square error of prediction (RMSEP), standard error of calibration (SEC), and ratio of performance to deviation (RPD) were calculated to assess the accuracy and robustness of the models.

PLS-DA was used to evaluate the effects of storage variables, including packaging material and storage form, on the discrimination of quality groups. The *mixOmics* package was utilized to construct and validate the PLS-DA models, incorporating VIP (Variable Importance in Projection) scores to identify the most influential factors. Score plots and loading plots were generated to visualize group separations and variable contributions, and performance was validated using a five-fold cross-validation procedure.

## 3. Results and Discussion

### 3.1. Peroxide Value

The POV is an indicator of the initial phase of lipid oxidation, reflecting the accumulation of hydroperoxides [21,22]. Although these primary oxidation products are relatively stable at the outset, they may eventually break down into secondary compounds that can adversely affect flavor and potentially impact health [23,24].

**Figure 1:**
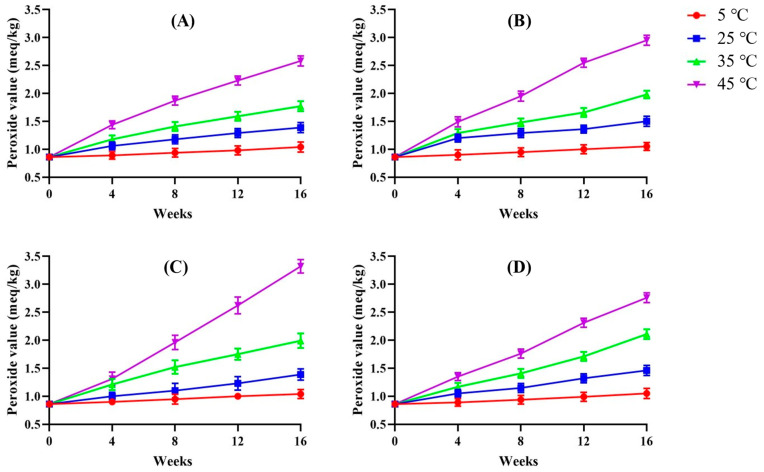
Peroxide value under different temperature conditions of Hong-Jam: raw form in PE (**A**), powder form in PE (**B**), raw form in glass (**C**), powder form in glass (**D**).

Figure 1 presents the changes in POV of Hong-Jam over a 16-week storage period under different temperature conditions. Overall, the POV increased as the storage temperature rose, indicating that elevated temperatures accelerated lipid oxidation. In samples stored in PE pouches, both the raw and powder forms showed the most pronounced increase in POV at 45 °C, followed by 35 °C, 25 °C, and 5 °C. Notably, the powder form exhibited consistently higher POVs than the raw form at all temperatures, suggesting that factors such as increased surface area and greater exposure to oxygen may have contributed to more rapid oxidation.

When comparing packaging materials, distinct patterns emerged depending on the physical state of the product. For the raw form, POV levels were higher in samples stored in glass bottles than in those kept in PE pouches at higher temperatures, implying that PE packaging provided relatively better protection against oxidation for the raw product. In contrast, the powder form stored in glass bottles showed slightly lower POVs at high temperatures compared to PE pouches, suggesting that the effectiveness of the packaging material may vary depending on the product’s physical form. These differences could be related to variations in oxygen permeability, headspace composition, moisture dynamics, and the inherent characteristics of each product state.

### 3.2. Acid Value

**Figure 2:**
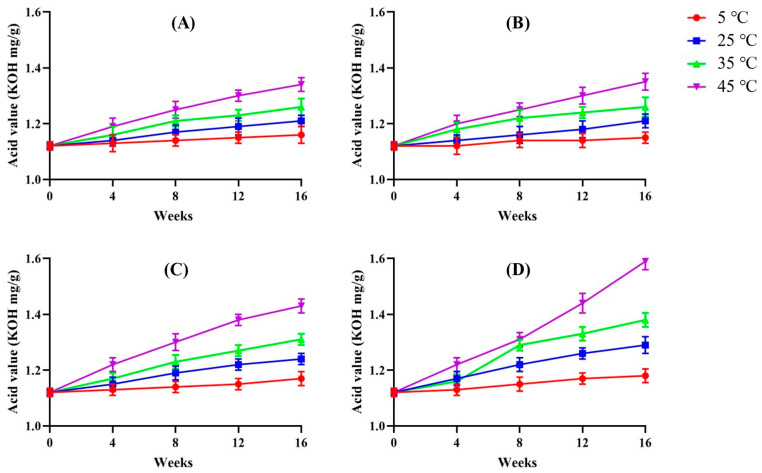
Acid value under different temperature conditions of Hong-Jam: raw form in PE (**A**), powder form in PE (**B**), raw form in glass (**C**), powder form in glass (**D**).

The AV reflects the concentration of free fatty acids, thereby indicating the degree of lipid hydrolysis [25]. As shown in Figure 2, the AV of Hong-Jam increased with rising storage temperature, closely mirroring the trend observed for POV. At 45 °C, all samples—regardless of packaging material or product form—exhibited the most pronounced AV increases, underscoring the strong temperature dependence of lipid hydrolysis. Elevated temperatures likely provide the activation energy necessary to break down triglycerides into free fatty acids at an accelerated rate, contributing to undesirable flavors, odors, and diminished nutritional quality [26,27,28].

In terms of product form, the powder samples consistently showed higher AV increments than their raw counterparts. This could be attributed to the increased surface area and enhanced exposure to hydrolytic conditions in powdered forms. The choice of packaging material also influenced the rate of hydrolysis. For example, at 45 °C and after 16 weeks of storage, powder samples in PE pouches generally exhibited lower AV values compared to those stored in glass containers, suggesting that PE may provide a more effective barrier against factors promoting hydrolysis. This difference could stem from variations in oxygen permeability, internal headspace conditions, or moisture interactions between the packaging material and the stored product.

Given that Hong-Jam contains a high proportion of unsaturated fatty acids, these results are consistent with their known susceptibility to oxidative and hydrolytic degradation at elevated temperatures [3,29]. By closely monitoring AV under different storage conditions, producers can better predict shelf life and develop strategies—such as low-temperature storage and appropriate packaging selection—to maintain lipid quality and minimize hydrolysis-induced quality losses.

### 3.3. Dependency of Temperature (D_T_)

**Figure 3:**
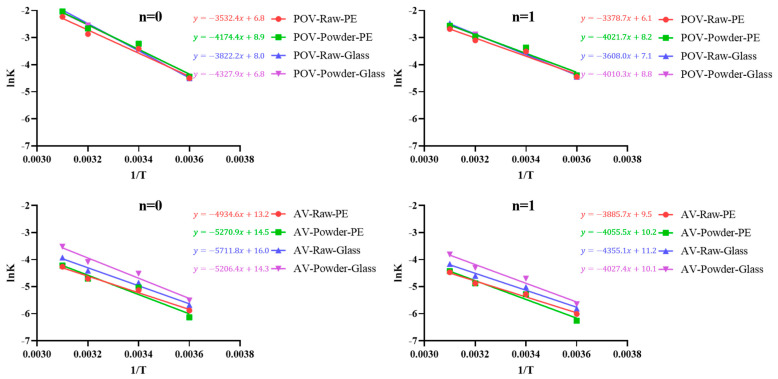
Dependency of temperature (DT) under different temperature conditions of Hong-Jam.

The relationship between the reaction rate constant (k) and temperature for changes in the POV and AV of Hong-Jam was derived from the Arrhenius equation (Figure 3). By plotting the natural logarithm (ln k) against the reciprocal of temperature (1/T), the temperature dependence of the rate constants under both zero-order (*n* = 0) and first-order (*n* = 1) reaction models was examined, providing insight into how lipid degradation kinetics vary with storage conditions [30,31].

Under the zero-order model, the reaction rate is assumed to be independent of the substrate concentration, implying that lipid degradation proceeds at a relatively constant rate once initiated. In contrast, the first-order model suggests that the rate is proportional to the remaining substrate concentration, making changes in reaction rates more sensitive to the amount of oxidizable lipids over time. By comparing the results from both models, it is possible to discern how packaging materials, product forms, and temperature interact to influence the degradation kinetics of Hong-Jam.

For POV, zero-order fits showed greater temperature dependence in raw samples stored in glass, while first-order fits revealed a stronger temperature response in powder—especially in PE pouches—where surface area and oxygen diffusion become limiting factors [32]. Thus, the reaction order changes the relative importance of packaging material and product form in determining temperature sensitivity. For AV, both models showed that PE pouches outperformed glass, yet first-order kinetics captured the progressive slowing of hydrolysis as substrates were depleted, again underscoring the protective role of PE over the full temperature range [33]. Because the R^2^ values for the first-order fits were consistently higher than those for the zero-order fits, we conclude that first-order kinetics better reflect the lipid-oxidation dynamics of Hong-Jam.

Overall, these results show that packaging, product form and temperature interact differently depending on reaction order, with glass bottles being more vulnerable to temperature-driven degradation than PE pouches in both oxidation (POV) and hydrolysis (AV) pathways. Generally, glass bottles provide lower oxygen- and water-vapor transmission rates than that of a PE pouch. However, the PE pouches were over-wrapped with an aluminum layer in our experiment that effectively upgraded their barrier performance. Therefore, faster deterioration in glass would be attributed primarily to this headspace oxygen load rather than intrinsic wall permeability. This scenario mirrors real consumer handling after the first opening and thus strengthens the practical relevance of our findings. On the other hand, the effects of controlled headspace oxygen have been reported to be effective [14]. When mealworm paste was stored at high-pressure under modified active packaging (MAP) with 0% O_2_, 70% CO_2_, and 30% N_2_, malondialdehyde formation was reduced by 40% after 28 days compared with air-packed controls [14].

### 3.4. $E_{a}$, $Q_{10}$, and Shelf Life

For POV and AV, the raw form generally exhibited lower activation energy ($E_{a}$) than the powder form, with the lowest $E_{a}$ observed for raw samples stored in PE pouches and the highest for powder samples stored in glass bottles under zero-order (*n* = 0) kinetics (Table 1). This suggests that, when the reaction rate is assumed independent of substrate concentration, raw samples—due to their higher moisture content and less dense structure—permit easier molecular mobility and thus require less energy for hydrolytic reactions to occur [34]. In contrast, powder samples, being more compact and lower in moisture, present a higher energy barrier.

Similar patterns persisted under first-order (*n* = 1) kinetics, where the reaction rate depends on the substrate concentration. Here, the interplay between substrate availability and reaction conditions becomes more pronounced. Notably, the coefficient of determination (R^2^) values for both POV and AV were generally slightly higher under first-order kinetics than under zero-order kinetics, indicating that the first-order model offered a better overall fit to the experimental data. While a higher R^2^ does not guarantee that the actual reaction mechanism strictly follows first-order kinetics, it does suggest that, within the conditions tested, the first-order model may more accurately capture the temperature dependence and degradation dynamics observed for Hong-Jam. Thus, from a statistical modeling standpoint—rather than a definitive mechanistic assertion—first-order kinetics may provide a more suitable framework for evaluating storage stability and temperature sensitivity.

Regarding $Q_{10}$, samples stored in glass bottles generally exhibited higher values than those in PE pouches, indicating a greater responsiveness to temperature changes [35]. In both reaction orders, the glass environment appeared more sensitive to thermal fluctuations, potentially due to factors such as residual oxygen or moisture interactions. For shelf life estimates, AV showed longer shelf life under PE pouches compared to glass bottles at both 5 and 25 °C, regardless of reaction order assumptions. This highlights the protective role of PE pouches in maintaining a more controlled microenvironment, slowing the progression of oxidative and hydrolytic processes, and thereby prolonging product quality [33]. Examining results from both zero- and first-order kinetics provides a more comprehensive perspective on how packaging materials, product forms, and temperature interact. Although the higher R^2^ values suggest that first-order kinetics may be statistically preferable for modeling these quality changes, it is essential to recognize that real food systems are complex, and the actual mechanistic pathways may not align perfectly with a single kinetic model.

### 3.5. Four-Way ANOVA Analysis

A four-way ANOVA was performed to quantify the single effects of packaging (A), product form (B), storage period (C) and temperature (D) as well as their two-, three- and four-way interactions on lipid-oxidation factors [36]. Comparing the magnitude of the F-values enabled us to gauge the relative contributions of factors of storage conditions and their interactions to the total variance in lipid-oxidation factors. The single effect of packaging is highly significant for both POV and AV, indicating that the choice of packaging material plays a crucial role in stabilizing these quality indicators (Table 2). In contrast, the effect of form (raw vs. powdered) is less significant for AV but notably significant for POV, suggesting that lipid oxidation is more sensitive to the product’s physical state. The larger surface area of the powdered form, which facilitates greater exposure to oxygen, likely contributes to its higher susceptibility to oxidation [37]. Additionally, both storage period and temperature show strong influences on POV and AV, reinforcing the concept that extended storage time and elevated temperature accelerate quality deterioration.

Interaction effects further refine these findings. The significant interaction between packaging and form (AB) implies that the effectiveness of a particular packaging type may differ depending on whether the product is in raw or powdered form. For example, powdered Hong-Jam stored in PE pouches may better maintain quality than when stored in glass, due to improved barrier properties and reduced oxidative stress. Similarly, the significant interaction between storage period and temperature (CD) highlights the necessity of controlling these factors simultaneously to minimize long-term quality loss.

When comparing the relative importance of factors, packaging (A) and form (B) emerge as dominant influences, both individually and through their interaction (AB). Although higher-order interactions (e.g., ABC, ABD, ACD and ABCD) are statistically significant, they generally have lower F-values, indicating that their effects, while present, are less critical than the primary and two-way interactions involving packaging and form.

In conclusion, this four-way ANOVA demonstrates that packaging type and product form are key determinants of Hong-Jam’s stability, with PE pouches and powdered forms offering particular advantages for maintaining POV and AV. While storage period and temperature also significantly affect quality, their roles appear secondary to the strong influence of packaging and form.

### 3.6. Moisture Content

In the previous sections (Section 3.1, Section 3.2, Section 3.3, Section 3.4 and Section 3.5), we measured and analyzed the quality changes of Hong-Jam during storage, focusing on POV and AV. In this section, we measured additional secondary quality indicators such as moisture, pH, and color in addition to the quality indicators used in the previous sections, and analyzed the effects of these indicators on the main quality indicators such as POV and AV.

The initial moisture content of Hong-Jam was relatively low due to the freeze-drying process and subsequent frozen storage before use (Table 3). Over the storage period, moisture content increased from approximately 2.87% to 4.05%, indicating that even under controlled conditions, gradual moisture uptake can occur. However, no statistically significant differences were observed between different packaging types or product forms. This suggests that, under the given experimental conditions, neither packaging material nor product form substantially influenced moisture absorption patterns. Similar findings were reported by [38], who observed increased moisture content in cricket powder as storage time and temperature rose, underscoring the general tendency of dried products to gradually gain moisture over extended storage periods.

Moisture content is critical for maintaining the quality and safety of dried foods. Increases in moisture can accelerate enzymatic reactions and lipid oxidation, and potentially support microbial growth, all of which can reduce product stability and shelf life [39]. Elevated moisture levels may also compromise sensory properties and degrade nutrients, such as vitamins and proteins, thereby diminishing the overall quality and nutritional value of the product. Although the increments in moisture content within this study were relatively modest, their steady upward trend highlights the importance of monitoring moisture as a quality factor. Even minor increases can serve as early indicators of potential quality deterioration over time. Thus, the assessment of moisture content may be employed as part of a comprehensive strategy to maintain the long-term storage stability and quality of Hong-Jam.

### 3.7. pH

The initial pH of Hong-Jam was approximately 7.27 and remained within a narrow range of 7.01 to 7.27 throughout the storage period, as shown in Table 4. Although a gradual decline in pH was observed over time, there were no statistically significant differences (*p* < 0.05) among the various packaging types or product forms. This decrease in pH is likely linked to the release of free fatty acids formed through lipid oxidation, as supported by the corresponding increase in acid value [40].

The interplay between moisture uptake and pH reduction further illustrates the complexity of quality changes during storage. As moisture content increases, it can facilitate lipid oxidation by promoting the formation of free radicals [39]. The resulting oxidation products, including free fatty acids, contribute to an overall decrease in pH. Although the changes in moisture and pH were relatively subtle, their cumulative effect can influence the product’s sensory attributes, nutritional profile, and overall stability [41].

A reduction in pH often serves as an early indicator of lipid oxidation and can lead to quality deterioration in high-fat foods [42]. Beyond influencing flavor, aroma, and nutritional value, the increased acidity may also affect protein solubility and functionality. Under more acidic conditions, proteins may denature or precipitate, altering the texture and mouthfeel of the product [43]. Such changes are particularly relevant for dried, protein- and lipid-rich products like Hong-Jam, where maintaining protein integrity is critical for preserving quality.

In summary, the decline in pH observed in Hong-Jam during storage reflects the accumulation of free fatty acids due to lipid oxidation. While neither packaging type nor product form significantly affected pH trends, these findings underscore the importance of monitoring pH in conjunction with moisture and lipid oxidation parameters. Such integrated assessments can provide a more comprehensive understanding of product stability, guiding strategies to maintain the sensory and nutritional quality of Hong-Jam over extended storage periods.

### 3.8. Color

**Figure 4:**
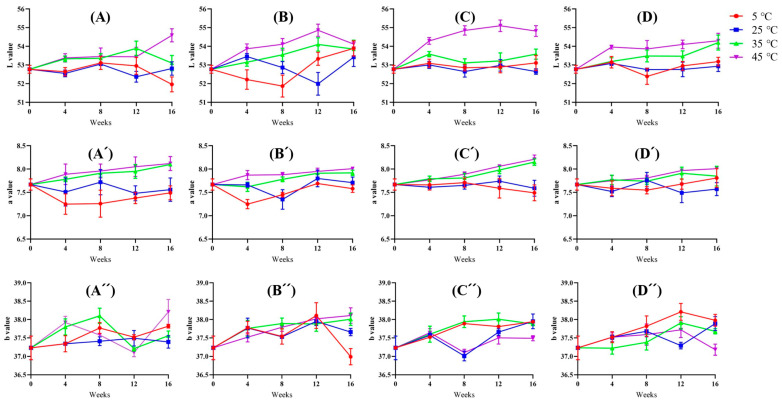
Changes in L (**A**–**D**), a (**A**′–**D**′), and b (**A**″–**D**″) values of Hong-Jam under different temperature conditions over 16 weeks: raw form in PE (**A**,**A**′,**A**″), powder form in PE (**B**,**B**′,**B**″), raw form in glass (**C**,**C**′,**C**″), powder form in glass (**D**,**D**′,**D**″).

The L value showed a slight upward trend during the 16-week storage period, with somewhat more pronounced increases observed at 45 °C, particularly in the raw form stored in glass bottles (Figure 4). However, these changes remained modest and were generally not statistically significant across most tested temperatures and conditions.

A similar, though less pronounced pattern was observed for a value, which exhibited minor fluctuations and a slight upward tendency at 45 °C without attaining statistical significance. This suggests that the red-green component of color was not strongly influenced by the tested storage conditions. In contrast, the b value remained notably stable, showing no appreciable variation regardless of temperature, product form, or packaging material. This stability in the yellow-blue component indicates that the pigments influencing b values maintained their resilience under the conditions examined.

In sum, while some observable chromaticity changes occurred at temperatures above 35 °C, these shifts could not be consistently attributed to a single reaction, nor did they appear reliably at other temperatures or conditions. In other words, the observed color changes were sporadic and lacked a clear trend. Such irregular and complex patterns reduce the utility of chromaticity as a reliable indicator of quality throughout storage. Based on these findings, it is unlikely that chromaticity can serve as a primary indicator of storage stability for Hong-Jam.

### 3.9. Principal Component 2D Biplot Graph

**Figure 5:**
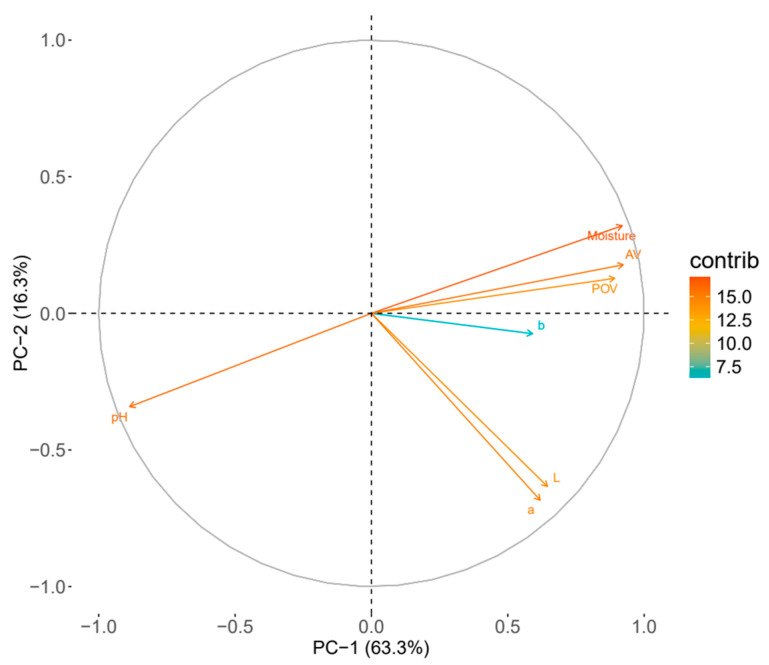
PCA biplot of the quality factors of Hong-Jam.

The PCA biplot in Figure 5 offers a comprehensive view of the interrelationships among quality factors in Hong-Jam, emphasizing how lipid oxidation indicators (POV and AV) interact with physicochemical parameters such as pH, moisture content, and color values. PC-1 and PC-2 explain approximately 63.3% and 16.3% of the total variance, respectively, accounting for around 79.6% of the dataset’s overall variability [36].

POV and AV strongly align along PC-1, indicating a close positive correlation between these two lipid oxidation indicators. Moisture content vectors project in a similar direction to POV and AV, suggesting that increased moisture may be associated with higher levels of lipid oxidation. In contrast, pH vectors extend in nearly the opposite direction, implying a negative correlation with oxidation indicators. Regarding color parameters, the b value (yellow-blue axis) shows a particularly notable positive association with POV and AV. While L and a values also contribute, b appears more distinctly aligned with the oxidation-related axis. This suggests that as lipid oxidation progresses, shifts in certain color components—especially yellow hues—may occur. This PCA-based insight implies that moisture and pH could serve as indirect markers for monitoring lipid oxidation.

### 3.10. Pearson Correlation Coefficient

Moisture content (M.C.) showed a strong positive correlation with both POV and AV (Table 5), suggesting that increased moisture may accelerate lipid oxidation, thereby elevating POV and AV levels. This finding is consistent with the PCA biplot (Figure 5), where moisture content is grouped closely with lipid oxidation indicators. Furthermore, M.C. displayed a negative correlation with pH, implying that samples with higher moisture content tended to have lower pH values, likely due to the accumulation of acidic oxidation byproducts. The strong negative correlations between pH and both POV and AV reinforce the notion that as lipid oxidation progresses, pH decreases as well a finding also consistent with the PCA biplot orientation of pH opposite to the oxidation vectors.

Color indices exhibited moderate positive correlations with POV and AV, with the b-value showing correlations of r = 0.41 (POV) and r = 0.43 (AV). These results suggest that as lipid oxidation progresses, some degree of yellowing may occur, supporting the PCA findings. However, the moderate magnitude of these correlations indicates that color indices alone do not provide sufficient predictive power to replace direct lipid oxidation measures.

In summary, the Pearson correlation analysis corroborates the relationships inferred from the PCA biplot, confirming that moisture content, pH, and color indices are linked to lipid oxidation in Hong-Jam. Yet, while these physicochemical factors mirror oxidation trends, their correlation strengths (particularly for color metrics) are not high enough to serve as standalone quality indicators. Among them, moisture content and pH appear most closely associated with oxidation, offering supplemental insights into product stability. Color changes may serve as indirect supportive indicators, particularly for visual quality assessment but are not robust predictors on their own. These findings highlight the value of integrating multiple parameters rather than relying solely on indirect measures when monitoring lipid oxidation and overall quality.

### 3.11. PLSR and PLS-DA

**Figure 6:**
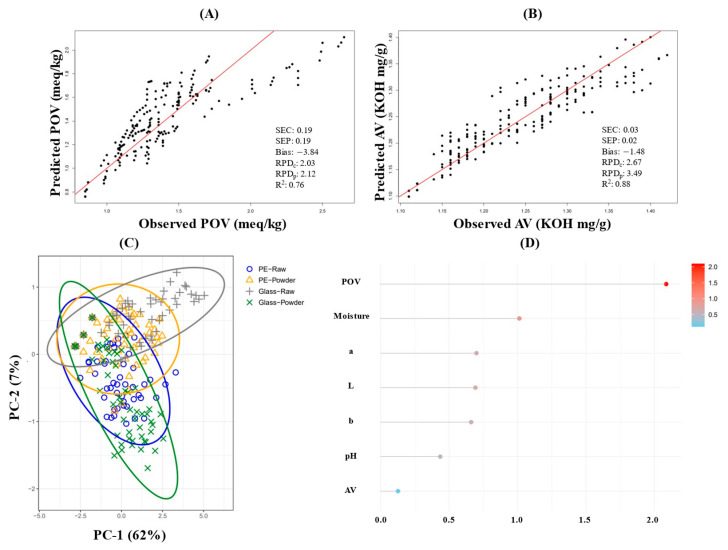
PLSR-based predictive model for peroxide value (POV): observed vs. predicted values (**A**), PLSR-based predictive model for Acid value (AV): observed vs. predicted values (**B**), PLS-DA score plot: group discrimination of Hong-Jam by packaging and product form (**C**), variable importance in projection (VIP) score plot: key influencing factors for Hong-Jam quality factors (**D**).

The correlation between the observed and the predicted lipid oxidation indices, peroxide value (POV) and acid value (AV), assessed via partial least squares regression (PLSR), is illustrated in Figure 6. For POV, the moderate predictive capability was indicated by an R^2^ of 0.76, along with identical calibration and prediction errors (SEC and SEP both 0.19) and a notable bias (−3.84), suggesting systematic underestimation. In contrast, AV showed higher predictive accuracy, characterized by minimal errors (standard error of calibration, SEC = 0.03; standard error of prediction, SEP = 0.02), a smaller bias (−1.48), and a higher R^2^ value of 0.88, reflecting consistently predictable behavior across storage conditions [44]. These differences underscore the limitation of linear PLSR modeling for POV, likely due to the non-linear oxidation dynamics, increased surface area exposure in powdered forms, and heightened sensitivity to temperature fluctuations. Future improvements could include adopting non-linear analytical approaches or integrating supplementary parameters such as moisture and pH.

Multivariate PLS-DA further clarified these quality distinctions by forming clear clusters according to packaging material and product form. Specifically, PE-packaged raw samples exhibited consistently stable quality, clearly differentiated from the more vulnerable powdered and glass-packaged samples. The variable importance (VIP) analysis reinforced these findings, identifying POV and moisture content (VIP scores > 1.0) as the most influential parameters affecting the Hong-Jam quality [45,46]. This result was consistent with earlier PCA and Pearson correlation analyses, highlighting moisture’s significant role in peroxide formation.

Collectively, the combined insights from PLSR and PLS-DA emphasize the necessity of integrating multiple analytical indicators for accurate quality prediction and management of Hong-Jam. While AV provides reliable predictive modeling suitable for streamlined quality control, direct experimental measurement of POV, particularly under challenging conditions, remains essential to ensure model accuracy and reliability.

## 4. Conclusions

This study demonstrates that the storage stability of Hong-Jam is primarily influenced by storage temperature, product form, and packaging type. Lipid oxidation indicators increased markedly above 25 °C, especially in powdered products, highlighting their effectiveness as primary quality markers. PE pouches consistently slowed these increases and thus outperformed glass bottles, a benefit particularly evident for powdered forms. Moisture content and pH are closely correlated with POV and AV; although not sufficient to replace these markers, they serve as practical supplementary indicators of product condition. Multivariate analyses (PCA, PLSR, PLS-DA) supported these relationships, identifying POV and moisture as the variables for best distinguishing the ideal storage conditions. Based on these findings, refrigerated storage (approximately 5 °C) is recommended whenever feasible, as it can extend the shelf life of Hong-Jam more than two-fold compared to storage at room temperature above 25 °C, approximately 58 months for raw products and 55 months for powder forms. This low-temperature strategy effectively reduces lipid oxidation and associated quality deterioration, potentially lowering costs and enabling producers to consistently deliver high-quality insect-based products to consumers. The outcomes of this research thus offer practical guidance to the food industry for improved product quality and enhanced logistical flexibility. Also, by offering a comprehensive map of the factors that govern stability, this work lays the groundwork for robust quality-management practices that meet the growing demand for sustainable, functional insect-based foods. Besides storage temperature, the effect of relative humidity on the shelf life of Hong-Jam is warranted for predicting the shelf life of insect food under actual distribution conditions.

## Figures and Tables

**Table 1:** Activation energy $\left(E_{a}\right)$, average $Q_{10}$, and shelf life for two quality factors of Hong-Jam.

RO *	QF *	Packaging	Condition	$E_{a}$ *	$Q_{10}$	SL5 *	SL25 *	$R^{2}$ *
*n* = 0	POV *	PE	Raw	7015	1.76	3850	1312	0.9947
			Powder	8290	1.83	8802	2912	0.9983
		Glass	Raw	7591	1.93	5578	2444	0.9815
			Powder	8595	1.78	4714	1744	0.9951
	AV *	PE	Raw	9800	1.50	26,077	8784	0.9936
			Powder	10,468	1.61	24,688	7319	0.9953
		Glass	Raw	11,344	1.55	26,077	8784	0.9943
			Powder	10,340	1.65	24,688	7756	0.9958
*n* = 1	POV	PE	Raw	7717	1.55	1774	696	0.9998
			Powder	8054	1.58	1688	604	0.9991
		Glass	Raw	8649	1.64	1774	696	0.9937
			Powder	7998	1.64	1688	632	0.9957
	AV	PE	Raw	6710	1.47	3000	1445	0.9952
			Powder	7987	1.58	3850	1312	0.9995
		Glass	Raw	7165	1.51	2461	1111	0.9936
			Powder	7964	1.58	2089	809	0.9979

* RO, reaction order; QF, quality factor; $E_{a}$, activation energy (cal/mol·K); SL5, shelf life at 5 °C (day); SL25, shelf life at 25 °C (day); $R^{2}$, Correlation coefficient; POV, peroxide value; AV, acid value.

**Table 2:** ANOVA of quality factors of Hong-Jam with different experimental factors.

Factor	F-Value							
	A*	B	C	D	A*B	A*C	A*D	B*C
POV **	5950.89 **	5613.90 **	18,834.50 **	2922.80 **	10,808.53 **	1411.92 **	10.56 **	1505.04 **
AV **	776.05 *****	1.17	4696.70 **	542.31 **	489.80 **	187.65 **	0.93	10.71 **
	B*D	C*D	A*B*C	A*B*D	A*C*D	B*C*D	A*B*C*D	
POV	11.30 **	251.39 **	1214.08 **	203.66 **	12.44 **	23.26 **	16.94 **	
AV	4.69 **	51.87 **	81.31 **	4.62 **	3.80 **	3.58 **	1.66	

* Four quality factors used in this study: packaging (A), product form (B), storage period (C), and temperature (D). ** POV, peroxide value; AV, acid value. ***** means statistically significant at *p* < 0.05 and *p* < 0.01, respectively.

**Table 3:** Moisture content (g/100 g Hong-Jam) under different temperature conditions of Hong-Jam.

Packaging	Product Form	Week	5 °C	25 °C	35 °C	45 °C
PE, Glass	Raw, Powder	0	2.87 ± 0.09 ^I^*	2.87 ± 0.09 ^F^	2.87 ± 0.09 ^G^	2.87 ± 0.09 ^I^
PE	Raw	4	3.15 ± 0.04 ^H^	3.18 ± 0.08 ^E^	3.16 ± 0.21 ^F^	3.21 ± 0.00 ^GH^
		8	3.31 ± 0.03 ^FG^	3.35 ± 0.02 ^DE^	3.32 ± 0.21 ^E^	3.29 ± 0.01 ^G^
		12	3.47 ± 0.04 ^E^	3.52 ± 0.04 ^CD^	3.58 ± 0.21 ^D^	3.51 ± 0.05 ^EF^
		16	3.51 ± 0.02 ^DE^	3.49 ± 0.07 ^CD^	3.63 ± 0.21 ^CD^	3.58 ± 0.06 ^DE^
	Powder	4	3.14 ± 0.04 ^H^	3.18 ± 0.08 ^E^	3.16 ± 0.05 ^F^	3.21 ± 0.00 ^GH^
		8	3.31 ± 0.03 ^FG^	3.35 ± 0.02 ^DE^	3.32 ± 0.02 ^E^	3.29 ± 0.01 ^G^
		12	3.47 ± 0.04 ^E^	3.52 ± 0.04 ^CD^	3.58 ± 0.04 ^D^	3.51 ± 0.05 ^EF^
		16	3.63 ± 0.05 ^CD^	3.59 ± 0.04 ^C^	3.71 ± 0.01 ^CD^	3.62 ± 0.04 ^CD^
Glass	Raw	4	3.22 ± 0.04 ^GH^	3.22 ± 0.08 ^e^	3.25 ± 0.21 ^EF^	3.19 ± 0.00 ^H^
		8	3.46 ± 0.03 ^E^	3.54 ± 0.02 ^C^	3.65 ± 0.21 ^CD^	3.59 ± 0.01 ^DE^
		12	3.76 ± 0.04 ^B^	3.87 ± 0.04 ^AB^	3.88 ± 0.21 ^B^	3.71 ± 0.05 ^B^
		16	3.89 ± 0.02 ^A^	4.02 ± 0.06 ^A^	4.05 ± 0.21 ^A^	3.99 ± 0.04 ^A^
	Powder	4	3.16 ± 0.04 ^H^	3.19 ± 0.08 ^E^	3.31 ± 0.21 ^E^	3.21 ± 0.02 ^GH^
		8	3.39 ± 0.03 ^EF^	3.46 ± 0.02 ^CD^	3.61 ± 0.21 ^CD^	3.44 ± 0.01 ^F^
		12	3.67 ± 0.04 ^BC^	3.78 ± 0.04 ^B^	3.75 ± 0.21 ^BC^	3.68 ± 0.05 ^BC^
		16	3.98 ± 0.00 ^A^	4.01 ± 0.11 ^A^	3.87 ± 0.03 ^B^	3.91 ± 0.05 ^A^

* Significant differences (*p* < 0.05) were indicated by means with different letters in the same column.

**Table 4:** pH value under different temperature conditions of Hong-Jam.

Packaging	Form	Week	5 °C	25 °C	35 °C	45 °C
PE, Glass	Raw, Powder	0	7.27 ± 0.01 ^A^*	7.27 ± 0.01 ^A^	7.27 ± 0.01 ^A^	7.27 ± 0.01 ^A^
PE	Raw	4	7.19 ± 0.01 ^BC^	7.15 ± 0.01 ^BC^	7.15 ± 0.00 ^BC^	7.14 ± 0.01 ^C^
		8	7.15 ± 0.02 ^CDE^	7.12 ± 0.02 ^CDE^	7.11 ± 0.02 ^D^	7.12 ± 0.01 ^CD^
		12	7.12 ± 0.01 ^DEF^	7.09 ± 0.01 ^DEFG^	7.09 ± 0.00 ^DEF^	7.07 ± 0.01 ^EF^
		16	7.04 ± 0.01 ^H^	7.05 ± 0.02 ^GHI^	7.05 ± 0.01 ^G^	7.04 ± 0.00 ^GH^
	Powder	4	7.20 ± 0.03 ^B^	7.19 ± 0.01 ^B^	7.18 ± 0.01 ^B^	7.19 ± 0.01 ^B^
		8	7.17 ± 0.02 ^BC^	7.15 ± 0.01 ^BC^	7.12 ± 0.02 ^CD^	7.13 ± 0.00 ^C^
		12	7.11 ± 0.01 ^EF^	7.08 ± 0.01 ^EFGH^	7.10 ± 0.01 ^DE^	7.02 ± 0.01 ^E^
		16	7.05 ± 0.01 ^GH^	7.04 ± 0.01 ^HI^	7.06 ± 0.01 ^FG^	7.01 ± 0.01 ^G^
Glass	Raw	4	7.18 ± 0.01 ^BC^	7.13 ± 0.02 ^CD^	7.15 ± 0.01 ^BC^	7.12 ± 0.00 ^D^
		8	7.16 ± 0.02 ^BCD^	7.09 ± 0.01 ^DEFG^	7.10 ± 0.00 ^DE^	7.08 ± 0.02 ^E^
		12	7.12 ± 0.01 ^DEF^	7.05 ± 0.02 ^GHI^	7.07 ± 0.02 ^EFG^	7.02 ± 0.01 ^HI^
		16	7.10 ± 0.01 ^F^	7.02 ± 0.01 ^I^	7.01 ± 0.01 ^H^	7.01 ± 0.01 ^I^
	Powder	4	7.20 ± 0.02 ^B^	7.18 ± 0.01 ^B^	7.15 ± 0.02 ^BC^	7.12 ± 0.01 ^CD^
		8	7.16 ± 0.02 ^BCD^	7.11 ± 0.01 ^CDEF^	7.12 ± 0.01 ^CD^	7.08 ± 0.01 ^E^
		12	7.12 ± 0.01 ^DEF^	7.07 ± 0.02 ^FGH^	7.07 ± 0.00 ^EFG^	7.04 ± 0.02 ^G^
		16	7.09 ± 0.01 ^FG^	7.06 ± 0.00 ^GHI^	7.06 ± 0.00 ^FG^	7.05 ± 0.01 ^FG^

* Significant differences (*p* < 0.05) were indicated by means with different letters in the same column.

**Table 5:** Pearson correlation coefficients of the quality factors of Hong-Jam.

	M.C.	pH	L	a	b	AV	POV
M.C. *							
pH	−0.89 ****						
L *	0.39 **	−0.39 **					
a *	0.36 **	−0.32 **	0.70 **				
b *	0.52 **	0.42 **	0.32 **	0.35 **			
AV *	0.91 **	−0.87 **	0.49 **	0.47 **	0.43 **		
POV *	0.84 **	−0.81 **	0.50 **	0.47 **	0.41 **	0.78 **	

* M.C., moisture content; L, lightness; a, redness; b, yellowness; AV, acid value; POV, peroxide value. ** means *p* < 0.01, indicating statistical significance. **** means statistically significant at *p* < 0.01.

## Data Availability

The original contributions presented in this study are included in the article. Further inquiries can be directed to the corresponding authors.
